# An Error-Adaptive Competition-Based Inverse Kinematics Approach for Bimanual Trajectory Tracking of Humanoid Upper-Limb Robots

**DOI:** 10.3390/biomimetics11040279

**Published:** 2026-04-17

**Authors:** Jiaxiu Liu, Zijian Wang, Hongfu Tang, Hongzhe Jin, Jie Zhao

**Affiliations:** School of Mechatronics Engineering, Harbin Institute of Technology, Harbin 150080, China; 22b308007@stu.hit.edu.cn (J.L.); 24b308009@stu.hit.edu.cn (Z.W.); tanghongfu@hit.edu.cn (H.T.); jzhao@hit.edu.cn (J.Z.)

**Keywords:** humanoid upper-limb robot, inverse kinematics, motion planning, trajectory tracking

## Abstract

Humanoid upper-limb robots are an important direction in biomimetic robotics, and inverse kinematics is a key technique for achieving human-like coordinated operation. However, existing inverse kinematics methods for bimanual trajectory tracking often suffer from high computational complexity and limited synchronization performance. To address this, this paper proposes an error-adaptive competition-based inverse kinematics (EAC-IK) approach for bimanual trajectory tracking of humanoid upper-limb robots. First, a unified modeling framework for the absolute tracking errors and synchronization errors of the two arms is established, and the end-effector task constraints are reformulated into a low-dimensional representation, thereby reducing the computational complexity of the original high-dimensional task mapping. Second, to enhance the coordination capability of bimanual operations, an error-adaptive competition mechanism is developed to regulate the weighting coefficients of the two arms online according to their error states. In addition, a virtual second-order command shaper is introduced at the joint level to reconstruct joint trajectories and suppress oscillations induced by input noise and the error-adaptive competition mechanism. Simulation and experimental results on a hyper-redundant humanoid upper-limb robot demonstrate that, compared with the zeroing neural-network-based inverse kinematics method, the proposed method achieves lower tracking and synchronization errors, as well as higher computational efficiency. In the circular trajectory-tracking experiment, the left-arm position and orientation tracking errors decrease from $1.60\times 10^{-3}\;m$ and $4.72\times 10^{-3}\;\mathrm{rad}$ to $0.70\times 10^{-3}\;m$ and $0.95\times 10^{-3}\;\mathrm{rad}$, respectively, while the synchronization error decreases from $1.96\times 10^{-3}$ to $1.30\times 10^{-3}$. In addition, the average algorithm runtime decreases from 0.82ms to 0.63ms.

## 1. Introduction

Humanoid upper-limb robots are currently an important research direction in the field of biomimetics. They typically consist of a torso and two arms, characterized by high degrees of freedom (DOFs) and strongly coupled multi-branch kinematic structures. Owing to these characteristics, such robots exhibit significant potential in complex manipulation tasks, including bimanual cooperative manipulation [1], precision assembly [2], and coordinated object transportation [3,4]. From a biomimetic perspective, research on humanoid upper-limb robots is concerned not only with constructing mechanical systems that possess human-like morphological characteristics, but also with enabling robots to achieve upper-limb coordination capabilities close to those of humans through the design of motion planning and control methods. When performing tasks such as bimanual carrying and fine manipulation, humans typically rely on coordinated cooperation between the torso and both arms to maintain synchronized arm motion while ensuring the operating accuracy of both end-effectors. However, although such bimanual coordination can significantly improve operational flexibility and task performance, it also imposes higher demands on inverse kinematics algorithms. Specifically, the algorithm must not only ensure accurate tracking of individual task objectives for each arm, but also suppress the relative errors between the two arms throughout the motion process to maintain synchronization consistency [5].

Regarding inverse kinematics of robotic systems, extensive research has been conducted from various perspectives. Among these, a representative class of approaches is the unified inverse kinematics strategy based on the Jacobian pseudoinverse, which treats all robot joints as a single system and stacks all end-effector tasks into a unified formulation. By constructing a high-dimensional Jacobian matrix and incorporating null-space projection, these methods enable multi-task coordination [6,7]. As a representative approach, the damped least-squares technique can alleviate singularity issues to some extent and enable effective computation of joint trajectories for redundant manipulators, and has been widely applied in trajectory-tracking control of industrial robotic arms [8,9]. However, for complex systems with high DOFs and multiple end-effector branches, the dimensionality of the Jacobian matrix increases significantly, leading to a rapid growth in the computational burden of pseudoinverse operations.

Another important class of methods is optimization-based inverse kinematics and motion generation. In these methods, multiple task requirements, including joint-limit constraints [10,11], obstacle avoidance [12,13], manipulability optimization [14], and contact-force or interaction-force regulation [15,16], are formulated as constraints and/or cost terms within a unified optimization framework. By leveraging techniques such as quadratic programming (QP) [10] and model predictive control (MPC) [17], these methods exhibit strong flexibility in handling complex task scenarios. Building upon this idea, whole-body control has become an important control framework for high-degree-of-freedom robotic systems, enabling coordinated control of the torso, dual arms, and mobile base to achieve integrated whole-body motion control [18]. For example, research [19] formulated the whole-body control problem of a humanoid robot as a QP problem, achieving accurate end-effector tracking while satisfying joint-velocity constraints and obstacle-avoidance constraints. One study [20] introduced MPC into the whole-body control of a mobile manipulator, obtaining control inputs that satisfied given constraints through receding-horizon optimization. However, although these optimization-based methods offer strong flexibility in handling multiple constraints and complex tasks, QP or MPC approaches for humanoid robots, which are typically high-dimensional systems operating at high control frequencies, usually require repeated online optimization and, thus, entail a relatively high computational burden [21]. In particular, MPC methods often rely on model linearization and receding-horizon optimization, which not only further increase the online computational cost but may also introduce additional approximation errors. Moreover, those optimization-based methods mainly focus on overall system coordination and constraint satisfaction, while paying relatively limited attention to the relative motion relationship required in bimanual cooperative tasks.

In summary, existing inverse kinematics methods, whether based on Jacobian-matrix-based unified solving strategies or optimization-based control frameworks, mainly focus on single-arm tracking accuracy or overall motion feasibility, while the synchronization consistency between the two arms is not explicitly addressed. In bimanual cooperative manipulation of rigid objects, the synchronization tracking accuracy between the two arms plays a critical role in system safety and operational quality. If the tracking errors of the two arms are inconsistent, additional internal contact forces may be generated at the interfaces between the end-effectors and the target object, potentially leading to object damage or task failure [22]. Therefore, several studies have introduced synchronization constraints or error coupling terms to enhance consistency between the two arms [23,24,25]. For example, P. Yu et al. modeled end-effector pose constraints using dual quaternions and achieved synchronous tracking control of high-DOF dual-robot systems by coupling individual arm tracking errors with inter-arm synchronization errors [23]. However, this method relies on empirically tuned fixed synchronization gains, making it difficult to achieve a dynamic balance between synchronization performance and tracking accuracy when task complexity varies or when error distributions become asymmetric.

**Figure 1:**
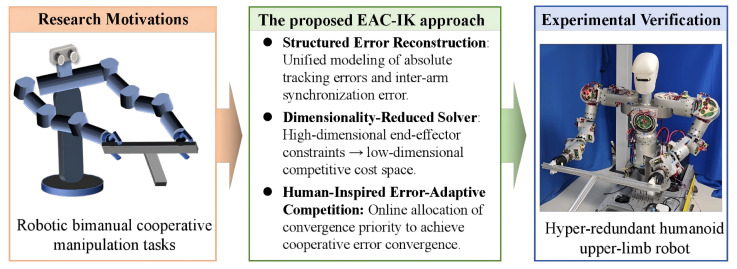
Overview of the proposed EAC-IK approach for humanoid upper-limb robot.

To address the problems of high computational complexity and insufficient bimanual synchronization in existing inverse kinematics methods, this paper proposes the EAC-IK approach for bimanual trajectory tracking of humanoid upper-limb robots. Specifically, by structurally reconstructing the bimanual end-effector task constraints, the coordinated bimanual objective is embedded into a low-dimensional constraint space, thereby reducing the dimensionality of inverse kinematics solving. Meanwhile, an error-state-based adaptive competition mechanism is introduced to regulate the contribution of the two arms online. This mechanism is motivated by the human ability to dynamically adjust bilateral participation in response to task states and coordination demands. As illustrated in Figure 1, the proposed EAC-IK approach generates joint-space trajectories in real time from the given end-effector pose trajectories. The main contributions of this paper are summarized as follows:

(1) A new EAC-IK method is proposed for humanoid upper-limb robots. It establishes a unified framework for the absolute tracking and synchronization errors of the dual arms and incorporates an error-adaptive competition mechanism to achieve high-precision end-effector tracking while maintaining bimanual synchronization.
(2) The proposed method structurally reformulates the end-effector task constraints of the dual arms, transforming the high-dimensional task mapping in conventional unified modeling into a low-dimensional constraint form, thereby enabling motion generation in a reduced-dimensional space.
(3) The effectiveness and superiority of the proposed method are demonstrated through comprehensive simulations and experimental validations on a hyper-redundant humanoid upper-limb robot platform. This constitutes another contribution of this paper.

The remainder of this paper is structured as follows: Section 2 presents the kinematic model of the humanoid upper-limb robot and provides the problem formulation. Section 3 describes the proposed EAC-IK, along with a comprehensive convergence analysis. Section 4 validates the effectiveness of the proposed method through numerical simulations. Section 5 reports experimental results obtained on a physical humanoid upper-limb robot platform. Finally, Section 6 concludes the paper.

## 2. Kinematic Modeling and Problem Formulation

Consider a class of humanoid upper-limb robots whose overall structure consists of a torso and two arms. Let the joint vector of the torso be denoted by $\theta_{b}\in R^{n_{b}}$ and the joint vectors of the left and right arms be denoted by $\theta_{k}\in R^{n_{a}},k\in \left\{1,2\right\}$, where k=1 represents the left arm and k=2 represents the right arm. The values of $n_{b}$ and $n_{a}$ represent the numbers of joints of the torso and the arm, respectively.

The end-effector pose vector of the *k*-th arm in Cartesian space is defined as

(1) $$Y_{k}=\left[\begin{array}{c} p_{k}\\ \phi_{k} \end{array}\right]\in R^{6}$$

where $p_{k}\in R^{3}$ denotes the position vector and $\phi_{k}\in R^{3}$ denotes the orientation vector. The mapping from the joint space to the task space of the robot is given by the following forward kinematics equation

(2) $$Y_{k}=f_{k}\left(q_{k}\right)$$

where $q_{k}=\left[\begin{array}{c} \theta_{b}\\ \theta_{k} \end{array}\right]\in R^{n_{b}+n_{a}}$ is the augmented joint vector associated with the *k*-th arm.

Furthermore, by differentiating (2) with respect to time and applying the chain rule, the velocity kinematic relationship of the *k*-th arm end-effector can be obtained as

(3) $${\dot{Y}}_{k}=J_{k}{\dot{q}}_{k}$$

where $J_{k}$ denotes the augmented Jacobian matrix corresponding to the *k*-th arm, given by

(4) $$J_{k}=\frac{\partial f_{k}}{\partial q_{k}}=\left[\begin{array}{cc} J_{2}^{k} & J_{1}^{k} \end{array}\right]\in R^{6\times (n_{b}+n_{a})}$$

with

$$J_{2}^{k}=\frac{\partial f_{k}}{\partial \theta_{b}}\in R^{6\times n_{b}},\;J_{1}^{k}=\frac{\partial f_{k}}{\partial \theta_{k}}\in R^{6\times n_{a}}.$$

Accordingly, (3) can be equivalently expanded as

(5) $${\dot{Y}}_{k}=J_{2}^{k}{\dot{\theta }}_{b}+J_{1}^{k}{\dot{\theta }}_{k}.$$

In (5), $J_{2}^{k}{\dot{\theta }}_{b}$ denotes the contribution of the torso joint motion to the pose velocity of the *k*-th end-effector, whereas $J_{1}^{k}{\dot{\theta }}_{k}$ denotes the contribution of the arm joint motion to the same end-effector. Since both terms quantify the motion of the *k*-th end-effector and are expressed in the robot base frame, they can be directly superimposed.

It is evident that, in humanoid upper-limb robots, the torso joints act as a shared kinematic chain for both arms. Their motion simultaneously affects the two end-effectors, thereby introducing significant structural coupling into bimanual cooperative motion and further influencing the synchronization performance between the two arms. In practical applications, the left and right arms mainly undertake precise tool-manipulation tasks, whereas the torso joints are primarily responsible for overall posture adjustment, workspace expansion, and coordinated support of arm motion. Therefore, the torso should possess a certain degree of independent regulation and planning capability in humanoid upper-limb robot motion.

Based on the above considerations, this paper focuses on a task-decoupled inverse kinematics approach for humanoid upper-limb robots. Assuming the torso motion as a predefined time-varying quantity, the proposed method enables coordinated and synchronous motion generation of the left and right arms. To this end, the following end-effector tracking errors and synchronization errors are defined

(6) $$\begin{array}{c} E_{1}=Y_{1}^{r}-Y_{1}\in R^{6},\;E_{2}=Y_{2}^{r}-Y_{2}\in R^{6},\;E_{12}=E_{1}-E_{2}\in R^{6}. \end{array}$$

where $Y_{1},Y_{2}\in R^{6}$ denote the actual end-effector pose vectors of the left and right arms, respectively, while $Y_{1}^{r},Y_{2}^{r}\in R^{6}$ denote the corresponding desired end-effector pose vectors. In practical robotic applications, $Y_{1}^{r}$ and $Y_{2}^{r}$ are usually predefined manually or obtained from visual sensing information [26,27,28]. $E_{1}$ and $E_{2}$ represent the corresponding end-effector tracking errors of the left and right arms, while $E_{12}$ denotes the synchronization error between the two arms.

To uniformly measure position errors (m) and orientation errors (rad), which have different physical meanings and numerical scales, and to achieve a reasonable balance between them during the convergence process, a symmetric positive definite matrix $M\in R^{6\times 6}$ is introduced. Based on this matrix, the following three scalar error measures are defined.

(7) $$\varepsilon_{1}=\sqrt{E_{1}^{T}ME_{1}},\;\varepsilon_{2}=\sqrt{E_{2}^{T}ME_{2}},\;\varepsilon_{3}=\sqrt{E_{12}^{T}ME_{12}}.$$

Since M is symmetric positive definite, each $\varepsilon_{i}$ defines a weighted norm of the corresponding error vector. Therefore, the convergence of $\varepsilon_{1}$, $\varepsilon_{2}$, and $\varepsilon_{3}$ to zero is, respectively, equivalent to the convergence of $E_{1}$, $E_{2}$, and $E_{12}$ to zero. Accordingly, the objective of this paper is to achieve coordinated convergence of the absolute tracking errors of the left and right arms together with the inter-arm synchronization error.

It should be noted that the scalar indices are introduced only to construct a unified weighted error metric. Therefore, the compression of error information occurs only at the metric level and does not affect the subsequent control solution process. The directional information contained in the original vector errors is preserved throughout the inverse kinematics solution and controller design, and, thus, no loss of the directional information required for motion generation is caused.

The weighting matrix M is chosen in the following block-diagonal form

(8) $$M=\left[\begin{array}{cc} m_{p}I_{3} & 0\\ 0 & m_{a}I_{3} \end{array}\right],$$

where $I_{3}$ denotes the 3×3 identity matrix and $m_{p},m_{a}>0$ are the weighting coefficients associated with the position and orientation errors, respectively. This block-diagonal form is adopted because it enables separate weighting of translational and rotational errors and avoids introducing unnecessary position–orientation cross-coupling terms. Since no prior preference is imposed on any specific translational direction or rotational axis in the considered task, isotropic weighting is adopted within each subspace through $I_{3}$. Furthermore, $m_{p}$ and $m_{a}$ are task-dependent design parameters, and their relative magnitudes can be used to reflect different task preferences regarding positional accuracy and orientation alignment. For example, when orientation alignment is of greater importance, $m_{a}$ can be increased accordingly; when the task places more emphasis on end-effector positional accuracy, $m_{p}$ can be increased instead.

**Remark** **1.** *It should be noted that conventional inverse kinematics methods typically stack the bimanual task constraints into a single vector and compute joint commands via the pseudoinverse of a high-dimensional Jacobian matrix. In contrast, this paper explicitly introduces the inter-arm synchronization error $E_{12}$ in the IK formulation and performs a structured reformulation of the absolute tracking errors and the synchronization error of the two end-effectors, mapping them into three error-energy measures ($\varepsilon_{1}$, $\varepsilon_{2}$, $\varepsilon_{3}$). This reformulation not only explicitly incorporates the synchronization constraint for bimanual tracking, but also transforms the original 12-dimensional end-effector task constraints into a three-dimensional error-constraint form, thereby providing a more direct mathematical basis and structural foundation for introducing the error-competition mechanism and constructing a low-dimensional inverse kinematics solver.*

## 3. Error-Adaptive Competition-Based Inverse Kinematics

In this section, the overall architecture of the proposed method is first briefly presented. Then, the inverse kinematics solution strategy based on the adaptive error-competition mechanism and the virtual second-order command shaper are introduced, together with their corresponding convergence analyses.

### 3.1. Framework Overview

**Figure 2:**
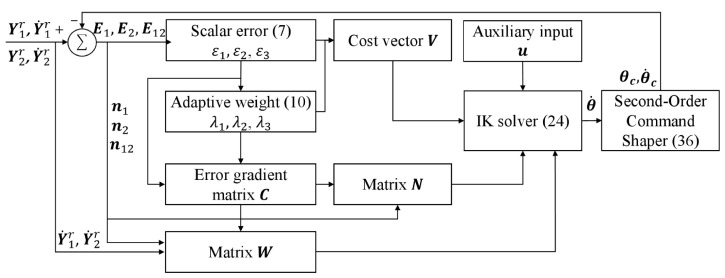
Block diagram of the proposed inverse kinematics method.

As shown in Figure 2, the proposed method is driven by the desired end-effector pose trajectory ($Y_{k}^{r},\;{\dot{Y}}_{k}^{r}$) and outputs the joint-angle command $\theta_{c}$ and the joint angular-velocity command ${\dot{\theta }}_{c}$ of the humanoid upper-limb robot. Specifically, the absolute end-effector tracking errors of the two arms and the dual-arm synchronization error are structurally reformulated into three scalar error measures ($\varepsilon_{1},\;\varepsilon_{2},\;\varepsilon_{3}$). Then, adaptive weighting coefficients ($\lambda_{1},\;\lambda_{2},\;\lambda_{3}$) are introduced to construct a three-dimensional error cost vector V, which characterizes the relative convergence priorities of different error terms and serves as the basis for the error-competition regulation mechanism. Based on the designed policy generator, i.e., the IK solver in (24), the joint angular-velocity trajectory $\dot{\theta }$ is generated to enforce the convergence of the primary error, whereas the term u is introduced as an auxiliary input to encode secondary objectives (such as joint-limit avoidance) without interfering with the main tracking task. Finally, a virtual second-order command shaper is employed to smooth and reshape $\dot{\theta }$, yielding dynamically reconstructed joint commands ($\theta_{c},\;{\dot{\theta }}_{c}$) that can be directly sent to the robot for execution.

### 3.2. Error-Adaptive Competitive-Based Inverse Kinematics

As stated in (7), the control objective of this paper is to achieve the coordinated convergence of the absolute tracking errors of the left and right arms and the inter-arm synchronization error. Accordingly, a three-dimensional error cost vector V based on a softmax-function competition mechanism is introduced as follows

(9) $$V=\left[\begin{array}{c} V_{1}\\ V_{2}\\ V_{3} \end{array}\right]=\left[\begin{array}{c} \lambda_{1}\varepsilon_{1}\\ \lambda_{2}\varepsilon_{2}\\ \lambda_{3}\varepsilon_{3} \end{array}\right]\in R^{3},$$

where $\lambda_{i}$ (i=1,2,3) denotes the adaptive weighting coefficient, which dynamically allocates the convergence priority according to the relative magnitudes of the three error measures. To construct the competition mechanism, the weights are defined using a softmax function as follows

(10) $$\lambda_{i}=\frac{\exp(\gamma \varepsilon_{i})}{\exp\left(\gamma \varepsilon_{1}\right)+\exp\left(\gamma \varepsilon_{2}\right)+\exp\left(\gamma \varepsilon_{3}\right)},$$

where γ>0 is used to regulate the competition intensity. A larger value of γ leads to a sharper weight distribution, in which the error component with the largest energy is assigned a higher weight, while the remaining weights are relatively suppressed. Obviously, the weighting coefficients $\lambda_{i}$ satisfy the following relations:

(11) $$\sum_{i=1}^{3}\lambda_{i}=1,\;\lambda_{i}\in \left(0,1\right)$$

It is worth noting that the competitive cost vector V unifies the three objectives, namely the absolute tracking error of the left arm, the absolute tracking error of the right arm, and the inter-arm synchronization error, into a single framework. Through the adaptive weighting coefficients $\lambda_{i}$, this framework dynamically allocates the suppression priority among the error components at each instant. This mechanism is partly inspired by human bimanual coordination: when performing cooperative two-handed tasks, humans tend to focus more on the arm with a larger deviation, thereby adaptively redistributing control effort between the two arms. For example, when the right-arm error $\varepsilon_{2}$ is dominant, the softmax function (10) automatically increases $\lambda_{2}$, thereby amplifying the contribution of $V_{2}$ in the cost dynamics and accelerating the attenuation of this dominant error.

Next, the cost dynamics of the system are analyzed. By taking the time derivative of V, we obtain

(12) $$\dot{V}=C\;\left(\varepsilon_{1},\varepsilon_{2},\varepsilon_{3}\right)\;\dot{\varepsilon },$$

where $C\;\left(\varepsilon_{1},\varepsilon_{2},\varepsilon_{3}\right)$ denotes the error gradient matrix and $\dot{\varepsilon }$ represents the time-derivative vector of the error measures. Their explicit expressions are given as follows

(13) $$C\;\left(\varepsilon_{1},\varepsilon_{2},\varepsilon_{3}\right)=\frac{\partial V}{\partial \varepsilon }=\left[\begin{array}{ccc} \frac{\partial V_{1}}{\partial \varepsilon_{1}} & \frac{\partial V_{1}}{\partial \varepsilon_{2}} & \frac{\partial V_{1}}{\partial \varepsilon_{3}}\\ \frac{\partial V_{2}}{\partial \varepsilon_{1}} & \frac{\partial V_{2}}{\partial \varepsilon_{2}} & \frac{\partial V_{2}}{\partial \varepsilon_{3}}\\ \frac{\partial V_{3}}{\partial \varepsilon_{1}} & \frac{\partial V_{3}}{\partial \varepsilon_{2}} & \frac{\partial V_{3}}{\partial \varepsilon_{3}} \end{array}\right]\in R^{3\times 3},$$

(14) $$\dot{\varepsilon }=\left[\begin{array}{c} {\dot{\varepsilon }}_{1}\\ {\dot{\varepsilon }}_{2}\\ {\dot{\varepsilon }}_{3} \end{array}\right]\in R^{3\times 1}.$$

Since the softmax-based weighting mapping is smooth with respect to the error variables, the matrix C is continuous with respect to the error variables. If the error measures and their time derivatives are bounded, then the matrix C and the time derivative of the cost vector $\dot{V}$ also remain bounded. Therefore, the constructed cost dynamics are well defined and can be used for the subsequent stability analysis and controller design.

Next, the matrix C and the error derivative vector $\dot{\varepsilon }$ are analyzed separately.

First, to derive the gradient matrix C, the partial derivatives of the softmax-based weights with respect to the error energies are provided. For any i,j∈{1,2,3}, it follows that

(15) $$\frac{\partial \lambda_{i}}{\partial \varepsilon_{j}}=\left\{\begin{array}{cc} \gamma \;\lambda_{i}\left(1-\lambda_{i}\right), & j=i,\\ -\gamma \;\lambda_{i}\lambda_{j}, & j\neq i. \end{array}\right.$$

This expression indicates that when $\varepsilon_{i}$ increases, the corresponding weight $\lambda_{i}$ increases as well (since the derivative is positive for j=i), while the other weights are suppressed (as the derivative is negative for j≠i), which intuitively reflects the competitive allocation property of the softmax function.

Furthermore, since $V_{i}=\lambda_{i}\varepsilon_{i}$, it follows that

(16) $$\frac{\partial V_{i}}{\partial \varepsilon_{j}}=\left\{\begin{array}{cc} \gamma \;\lambda_{i}\left(1-\lambda_{i}\right)\varepsilon_{i}+\lambda_{i},\; & j=i,\\ -\gamma \;\lambda_{i}\lambda_{j}\;\varepsilon_{i},\; & j\neq i. \end{array}\right.$$

By substituting (16) into (13), the explicit expression of the matrix C can be obtained as

(17) $$\begin{array}{cc} \; & C=\left[\begin{array}{ccc} \lambda_{1}+\gamma \lambda_{1}\left(1\;-\;\lambda_{1}\right)\varepsilon_{1}\; & -\gamma \lambda_{1}\lambda_{2}\varepsilon_{1}\; & -\gamma \lambda_{1}\lambda_{3}\varepsilon_{1}\\ -\gamma \lambda_{2}\lambda_{1}\varepsilon_{2}\; & \lambda_{2}+\gamma \lambda_{2}\left(1\;-\;\lambda_{2}\right)\varepsilon_{2}\; & -\gamma \lambda_{2}\lambda_{3}\varepsilon_{2}\\ -\gamma \lambda_{3}\lambda_{1}\varepsilon_{3}\; & -\gamma \lambda_{3}\lambda_{2}\varepsilon_{3}\; & \lambda_{3}+\gamma \lambda_{3}\left(1\;-\;\lambda_{3}\right)\varepsilon_{3} \end{array}\right]. \end{array}$$

Second, the term $\dot{\varepsilon }$ is analyzed. For convenience of analysis, when $\varepsilon_{i}\neq 0$, the normalized gradient of the error is defined as

(18) $$\begin{array}{c} n_{1}=\frac{ME_{1}}{\varepsilon_{1}}\in R^{6\times 1},\;n_{2}=\frac{ME_{2}}{\varepsilon_{2}}\in R^{6\times 1},\;n_{12}=\frac{ME_{12}}{\varepsilon_{3}}\in R^{6\times 1}. \end{array}$$

Thus, we have

(19) $${\dot{\varepsilon }}_{1}=n_{1}^{⊤}{\dot{E}}_{1},\;{\dot{\varepsilon }}_{2}=n_{2}^{⊤}{\dot{E}}_{2},\;{\dot{\varepsilon }}_{3}=n_{12}^{⊤}{\dot{E}}_{12}.$$

Considering that ${\dot{E}}_{12}={\dot{E}}_{1}-{\dot{E}}_{2}$, the relationship between the derivatives of the scalar error measures (${\dot{\varepsilon }}_{1},{\dot{\varepsilon }}_{2},{\dot{\varepsilon }}_{3}$) and the derivatives of the error vectors (${\dot{E}}_{1},{\dot{E}}_{2}$) can be established as

(20) $$\left[\begin{array}{c} {\dot{\varepsilon }}_{1}\\ {\dot{\varepsilon }}_{2}\\ {\dot{\varepsilon }}_{3} \end{array}\right]=N_{E}\left[\begin{array}{c} {\dot{E}}_{1}\\ {\dot{E}}_{2} \end{array}\right].$$

Let $0_{1\times 6}$ denote a 1×6 zero vector, then $N_{E}$ is given by

(21) $$N_{E}=\left[\begin{array}{cc} n_{1}^{⊤} & 0_{1\times 6}\\ 0_{1\times 6} & n_{2}^{⊤}\\ n_{12}^{⊤} & -n_{12}^{⊤} \end{array}\right]\in R^{3\times 12}.$$

By combining (5), (6), (12) and (20), the system cost dynamics are ultimately rearranged into the following form

(22) $$\dot{V}=W-N\dot{\theta }$$

where $\dot{\theta }={\left[{\dot{\theta }}_{1}^{⊤},\;{\dot{\theta }}_{2}^{⊤}\right]}^{⊤}$ represents the joint angular velocity vector of the dual arms of the humanoid upper-limb robot. The matrices W and N are defined as

(23) $$\begin{array}{cc} \; & W=CN_{E}\left(\left[\begin{array}{c} {\dot{Y}}_{1}^{r}\\ {\dot{Y}}_{2}^{r} \end{array}\right]+\left[\begin{array}{c} J_{2}^{1}\\ J_{2}^{2} \end{array}\right]{\dot{\theta }}_{b}\right)\in R^{3\times 1},\\ \; & N=CN_{E}\left[\begin{array}{cc} J_{1}^{1} & 0_{6\times n_{a}}\\ 0_{6\times n_{a}} & J_{1}^{2} \end{array}\right]\in R^{3\times 2n_{a}}. \end{array}$$

where $0_{6\times n_{a}}$ denotes a $6\times n_{a}$ zero matrix.

From a system-theoretic perspective, the error dynamics in (22) essentially constitute a controllable system with $\dot{\theta }$ as the input and V as the state. Therefore, solving the joint trajectory command $\dot{\theta }$ for the dual arms of the humanoid upper-limb robot is, in essence, a stabilization control problem with respect to V. To drive V to zero and achieve cost minimization, the desired dynamics of V are specified as $\dot{V}\propto -V$. This implies that the evolution of V is always directed along its negative direction, thereby ensuring its gradual decay and eventual convergence to zero. Therefore, the requirement on V should essentially be interpreted as prescribed desired convergence dynamics.

In the following, the inverse kinematics relationship is formulated in the form of a theorem.

**Theorem** **1.** *The cost dynamics (22) of the system are known. Under the cost minimization principle, the following candidate constraint equation is chosen*

(24) $$\dot{\theta }=N^{+}\;\left(kV+W\right)+\left(I-N^{+}N\right)u$$

*where k>0 is a tunable gain parameter and $N^{+}$ denotes the pseudoinverse of N. The term $\left(I-N^{+}N\right)$ represents the null-space projection matrix of N. $u\in R^{2n_{a}\times 1}$ denotes an auxiliary input term used to incorporate secondary motion objectives, such as joint-limit avoidance, obstacle avoidance, and manipulability optimization, without affecting the primary tracking task. This term can be flexibly designed according to specific task requirements, as illustrated in refs. [29,30]. Under the proposed control law (24), i.e., the IK solver, the cost V converges to zero, which implies that $\varepsilon_{1}$, $\varepsilon_{2}$, and $\varepsilon_{3}$ asymptotically converge to zero, thereby ensuring the simultaneous suppression of the absolute tracking errors of both arms and the inter-arm synchronization error. Meanwhile, $\dot{\theta }$ converges to u in the null space, indicating that the joint motions are guided to satisfy environmental constraints and additional performance criteria without affecting the convergence of the primary task.*

**Remark** **2.** *In the proposed IK solver (24), the primary task is solved via the pseudoinverse of $N\in R^{3\times 2n_{a}}$; in contrast, conventional inverse kinematics methods typically require computing the pseudoinverse of a high-dimensional task Jacobian $J\in R^{12\times 2n_{a}}$. By reconstructing the primary task using low-dimensional error constraints, the proposed method substantially reduces the scale of online matrix operations and the complexity of pseudoinverse computation. Moreover, the competitive weights $\lambda_{i}$ embed the scheduling rule of “larger error, higher convergence priority” into the solver structure to ensure synchronized-tracking convergence of the primary task. The null-space term $\left(I-N^{+}N\right)u$ further accommodates secondary objectives (such as joint-limit avoidance or obstacle avoidance) without compromising the primary task.*

**Proof.**  First, we prove that V converges to zero. By substituting (24) into (22), it can be obtained that

(25) $$\dot{V}=W-N\;\left[N^{+}\;\left(kV+W\right)+\left(I-N^{+}N\right)u\right].$$

Noting that $NN^{+}=I$ and $N\left(I-N^{+}N\right)=0$, it follows that

(26) $$\dot{V}=-kV$$

It is evident that when k>0, the system is stable. Therefore, V converges to zero, which further implies that $\dot{V}$ also converges to zero.

Next, the convergence of $\varepsilon_{1}$, $\varepsilon_{2}$, and $\varepsilon_{3}$ is established. Since the weighting coefficients satisfy $\sum_{i=1}^{3}\lambda_{i}=1$, it follows that $\max_{i}\left\{\lambda_{i}\right\}\geq 1/3$. Let $S=\max\{\varepsilon_{1},\varepsilon_{2},\varepsilon_{3}\}$, and denote by $i^{*}$ the index corresponding to the maximum error. Then, it holds that

(27) $${∥V∥}_{\infty }\geq V_{i^{*}}=\lambda_{i}\;\varepsilon_{i^{*}}\geq \frac{1}{3}\;S.$$

Based on the above analysis, it has been shown that V converges to zero. Consequently, *S* also converges to zero, which implies that $\varepsilon_{1}$, $\varepsilon_{2}$, and $\varepsilon_{3}$ all converge to zero.

Finally, the convergence of $\dot{\theta }$ to u is established. Left-multiplying both sides of (24) by $\left(I-N^{+}N\right)$ yields

(28) $$\begin{array}{c} \left(I-N^{+}N\right)\dot{\theta }=\left(I-N^{+}N\right)N^{+}\left(kV+W\right)+\left(I-N^{+}N\right)\left(I-N^{+}N\right)u. \end{array}$$

Since $N^{+}NN^{+}=N^{+}$, it follows that

(29) $$\begin{array}{cc} \left(I-N^{+}N\right)N^{+}=0, & \;\left(I-N^{+}N\right)\left(I-N^{+}N\right)=I-N^{+}N. \end{array}$$

Therefore, (28) can be rewritten as

(30) $$\left(I-N^{+}N\right)\left(\dot{\theta }-u\right)=0$$

This equation indicates that the component of $\dot{\theta }$ in the null space coincides with u, i.e., the joint velocities strictly follow u within the null space without affecting the primary task. This completes the proof.    □

### 3.3. Virtual Second-Order Command Shaper

The inverse kinematics solver (24) is capable of dynamically generating the joint command θ and $\dot{\theta }$ based on the task-space commands $Y_{k}^{r}$ and ${\dot{Y}}_{k}^{r}$. However, in practical robotic applications, $Y_{k}^{r}$ and ${\dot{Y}}_{k}^{r}$ are often obtained through measurement or estimation using external sensors, such as vision and force sensors, and are, therefore, inevitably contaminated by noise. Although noise can be mitigated through image processing or signal filtering techniques, it is generally difficult to eliminate it completely, resulting in residual high-frequency components in θ and $\dot{\theta }$. In addition, the error-competition mechanism may also introduce command fluctuations during dynamic weight adjustment. If such commands are directly used as joint-level control inputs, they may induce joint trajectory oscillations or even excite mechanical vibrations, thereby degrading the smoothness and stability of robot motion.

To this end, a virtual second-order command shaper is introduced at the joint level to impose a realizable second-order dynamic constraint on the joint commands generated by the inverse kinematics solver. The shaper essentially acts as a dynamic constraint mapping, aiming to suppress high-frequency components in the joint commands and enhance trajectory smoothness. The proposed virtual second-order command shaping dynamics are defined as

(31) $${\ddot{\theta }}_{c}\left(t\right)+2\xi \omega \;{\dot{\theta }}_{c}\left(t\right)+\omega^{2}\theta_{c}\left(t\right)=\omega^{2}\theta \left(t\right)+2\xi \omega \;\dot{\theta }\left(t\right).$$

where θ(t) and $\dot{\theta }\left(t\right)$ denote the original joint commands obtained from the inverse kinematics solver (24), and $\theta_{c}\left(t\right)$ represents the shaped joint angles. The parameter ξ>0 is the damping ratio, and ω>0 is the characteristic frequency. This model constructs a second-order dynamic mapping that constrains the dynamic behavior of the shaped joint commands. From a physical perspective, it is equivalent to introducing a second-order system with virtual mass 1, damping 2ξω, and stiffness $\omega^{2}$ at the joint level, thereby effectively suppressing high-frequency noise in the joint trajectories.

By applying the Laplace transform to (31), the transfer function G(s) from θ to $\theta_{c}$ can be obtained as

(32) $$G\left(s\right)=\frac{\theta_{c}\left(s\right)}{\theta (s)}=\frac{\omega^{2}+2\xi \omega s}{s^{2}+2\xi \omega s+\omega^{2}}.$$

This transfer function indicates that the introduced virtual second-order dynamics essentially constitute a second-order low-pass mapping system. It preserves the overall trend of joint motion in the low-frequency range, while effectively attenuating noise and rapidly varying components in the high-frequency range.

It should be noted that the virtual second-order command shaper may introduce a certain dynamic lag while smoothing the input command. This lag is mainly reflected in phase delay and a slower transient response, and is closely related to the parameters ξ and ω. In general, a smaller ω is beneficial for enhancing the smoothing effect, but it also leads to a slower response; a larger ω, by contrast, helps improve the response speed. Meanwhile, variations in ξ also affect the smoothness of the transition process. In the motion-planning task considered in this paper, this effect mainly appears during transient stages when the command changes rapidly, while its influence on slowly varying or steady-state stages is relatively limited. Therefore, the parameter selection should balance trajectory smoothness and response speed.

To analyze the error characteristics introduced by (31), the shaping error is defined as

(33) $$e_{\theta }\left(t\right)=\theta_{c}\left(t\right)-\theta \left(t\right).$$

Based on (31) and (33), the error dynamics of the command shaper can be derived as

(34) $${\ddot{e}}_{\theta }+2\xi \omega {\dot{e}}_{\theta }+\omega^{2}e_{\theta }=-\ddot{\theta }.$$

By rewriting the above equation in the state-space form, we obtain

(35) $$\dot{x}=Ax+B\mu \left(t\right),\;x=\left[\begin{array}{c} e_{\theta }\\ {\dot{e}}_{\theta } \end{array}\right],$$

where

$$A=\left[\begin{array}{cc} 0 & I\\ -\omega^{2}I & -2\xi \omega \;I \end{array}\right],\;B=\left[\begin{array}{c} 0\\ I \end{array}\right],\;\mu \left(t\right)=-\ddot{\theta }.$$

The eigenvalues of A are given by $\lambda =-\xi \omega \pm \omega \sqrt{\xi^{2}-1}$. Hence, for ξ>0 and ω>0, all eigenvalues of A have strictly negative real parts, i.e., A is Hurwitz. Therefore, the error system is exponentially stable in the absence of input and is input-to-state stable with respect to the input μ(t).

Finally, by equivalently rewriting (31) in the state-space form, the final implementation of the virtual second-order command shaper can be obtained as

(36) $$\left\{\begin{array}{c} {\dot{X}}_{1}=X_{2},\\ {\dot{X}}_{2}=\omega^{2}\left(\theta -X_{1}\right)+2\xi \omega \left(\dot{\theta }-X_{2}\right). \end{array}\right.$$

where $X_{1}=\theta_{c}$ and $X_{2}={\dot{\theta }}_{c}$ denote the reconstructed joint-angle and joint-velocity trajectories, respectively, which are used to control the robot motion.

Based on the above derivations, the overall implementation procedure of the proposed method is summarized in Algorithm 1.
**Algorithm 1** Error-Adaptive Competition-Based Inverse Kinematics**Input:** Desired end-effector pose trajectories of the left and right arms, denoted by $Y_{1}^{r}\left(t\right),Y_{2}^{r}\left(t\right)$, respectively, and their velocities ${\dot{Y}}_{1}^{r}\left(t\right)$ and ${\dot{Y}}_{2}^{r}\left(t\right)$; task execution time *T*; parameters γ,k,ξ,ω.**Output:** Joint angle commands $\theta_{c}$; joint velocity commands ${\dot{\theta }}_{c}$.**Note:** Subscripts 1 and 2 denote the left and right arms, respectively, and the superscript *r* denotes the desired/reference quantity.     1:Initialize time t←0     2:**while** t<T **do**     3:   Compute the actual end-effector pose vectors $Y_{1}$ and $Y_{2}$ via forward kinematics (2)     4:   Compute tracking errors $E_{1}\leftarrow Y_{1}^{r}-Y_{1}$, $E_{2}\leftarrow Y_{2}^{r}-Y_{2}$, and synchronization error $E_{12}\leftarrow E_{1}-E_{2}$     5:   Compute scalar error measures $\varepsilon_{1},\varepsilon_{2}$, and $\varepsilon_{3}$ according to (7)     6:   Update adaptive competition weights $\lambda_{1},\lambda_{2}$, and $\lambda_{3}$ according to (10)     7:   Construct cost vector $V\leftarrow {\left[\lambda_{1}\varepsilon_{1},\;\lambda_{2}\varepsilon_{2},\;\lambda_{3}\varepsilon_{3}\right]}^{T}$     8:   Update gradient matrix C according to (17)     9:   Update matrices N and W according to (23)   10:   Compute joint velocity command by IK solver according to (24), $\dot{\theta }\leftarrow N^{+}\left(kV+W\right)+\left(I-N^{+}N\right)u$   11:   Update shaped joint commands $\theta_{c}$ and ${\dot{\theta }}_{c}$ using the virtual second-order command shaper (36)   12:**end while**

## 4. Numerical Simulation

This section validates the effectiveness of the proposed method (EAC-IK) through simulation experiments on a humanoid upper-limb robot and provides a quantitative performance comparison with representative methods proposed in recent years. The baseline method is the Zeroing Neural Network (ZNN) approach proposed in [31], which is a typical online solver for time-varying linear equations and has been widely used in robot inverse kinematics and real-time control applications.

### 4.1. Simulation Setup

**Figure 3:**
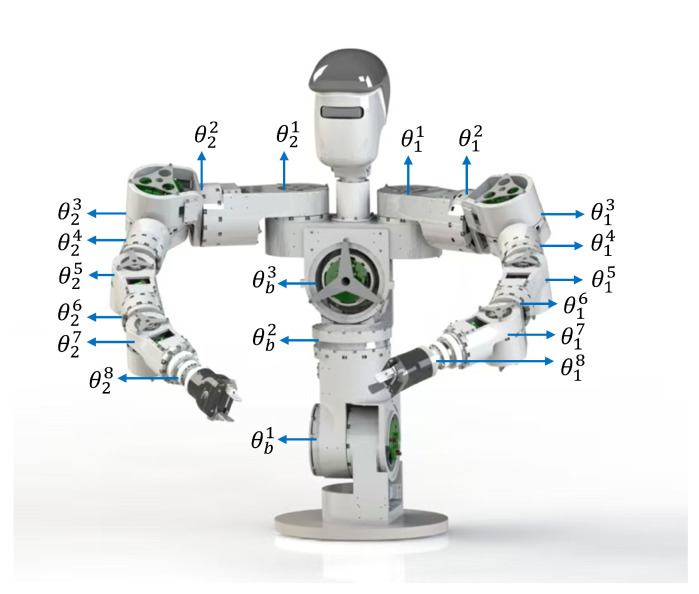
The humanoid upper-limb robot model used in this paper.

In this study, a 22-DOF humanoid upper-limb robot is adopted as the simulation and experimental validation platform. As shown in Figure 3, the robot consists of a three-DOF torso ($n_{b}=3$), two eight-DOF arms ($n_{a}=8$), and a three-DOF head. This configuration exhibits high kinematic redundancy and strong structural coupling while preserving sufficient dexterity for bimanual end-effector manipulation, thus providing a representative testbed for validating the effectiveness and synchronization performance of the proposed method under complex coupled kinematic conditions.

To systematically evaluate the performance of the proposed method in bimanual trajectory tracking, the desired end-effector pose trajectories of the left and right end-effectors are specified as follows

(37) $$Y_{1}^{r}\left(t\right)=\left[\begin{array}{c} 0.55+0.15\sin(0.5t)\\ -0.57+0.02\sin(0.5t)\\ 0.45+0.10\sin(t)\\ -\frac{\pi }{6}+\frac{\pi }{45}\sin\left(0.3t\right)\\ \frac{2\pi }{45}\sin\left(0.3t\right)\\ \frac{\pi }{45}\sin\left(0.3t\right) \end{array}\right],\;Y_{2}^{r}\left(t\right)=\left[\begin{array}{c} -0.55+0.15\sin(0.5t)\\ -0.57+0.02\sin(0.5t)\\ 0.45+0.10\sin(t)\\ -\frac{\pi }{6}+\frac{\pi }{45}\sin\left(0.3t\right)\\ \frac{2\pi }{45}\sin\left(0.3t\right)\\ \frac{\pi }{45}\sin\left(0.3t\right) \end{array}\right].$$

where $Y_{1}^{r}\left(t\right)$ and $Y_{2}^{r}\left(t\right)$ denote the desired end-effector pose trajectories in the task space for the left and right arms, respectively, including both position and orientation components. To emulate noise disturbances in practical trajectory inputs, zero-mean Gaussian noise with a signal-to-noise ratio of 55 is injected into $Y_{1}^{r}$ and $Y_{2}^{r}$. The torso trajectory is specified as a predefined time-varying function $\left[\frac{\pi }{36}+\frac{\pi }{36}\sin\left(0.2t\right);\frac{\pi }{36}\sin\left(0.2t\right);\frac{\pi }{60}\sin\left(0.1t\right)\right]$ rad.

The parameter settings of the proposed method are given as follows. For the dual-arm trajectory-tracking task considered in this paper, the position-error weight and orientation-error weight are chosen as $m_{p}=1$ and $m_{a}=0.5$, respectively. These values are empirically selected to prioritize positional tracking accuracy while maintaining sufficient orientation constraints for stable convergence. Accordingly, M=diag{1,1,1,0.5,0.5,0.5}. The remaining parameters are set as: γ=100, and k=150 in (24), ξ=2 and ω=30 in (36). A fifth-order Adams–Moulton method is employed for discretization. The sampling period is set to 0.001 s, and the total motion duration is 40 s.

### 4.2. Simulation Results and Analysis

To systematically evaluate the performance of the proposed EAC-IK method, absolute tracking accuracy, synchronization performance, and computational time are selected as evaluation metrics. The absolute tracking accuracy includes the left-arm position tracking error $∥e_{1}^{p}∥$, left-arm orientation tracking error $∥e_{1}^{a}∥$, right-arm position tracking error $∥e_{2}^{p}∥$, and right-arm orientation tracking error $∥e_{2}^{a}∥$, where ∥·∥ denotes the $\ell_{2}$-norm. The bimanual synchronization performance is assessed by the synchronization error index $\varepsilon_{3}$ defined in (7), where a smaller $\varepsilon_{3}$ indicates better bimanual synchronization.

**Figure 4:**
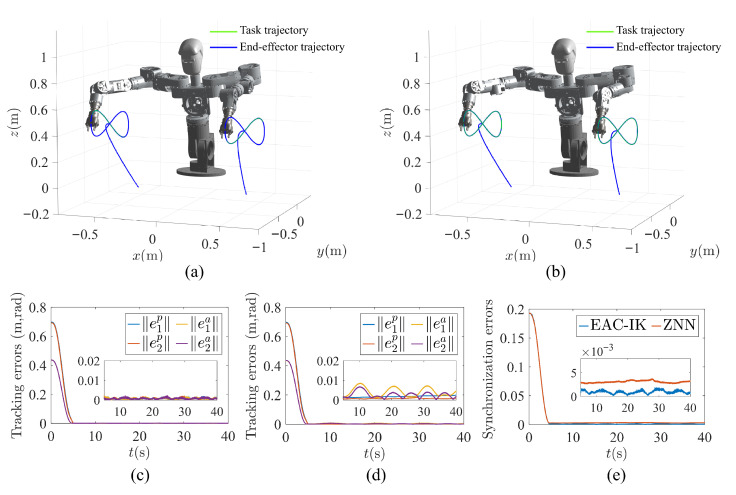
Comparison of robot trajectory-tracking results between EAC-IK and ZNN. (**a**) End-effector trajectory obtained by EAC-IK. (**b**) End-effector trajectory obtained by ZNN. (**c**) Pose tracking errors of EAC-IK. (**d**) Pose tracking errors of ZNN. (**e**) Comparison of synchronization errors.

**Figure 5:**
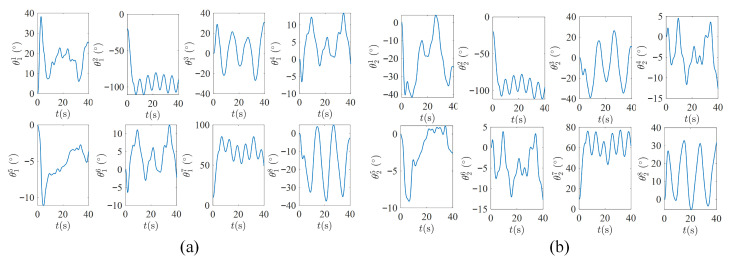
Joint trajectories of both arms generated by the EAC-IK method. (**a**) Left-arm joint trajectories. (**b**) Right-arm joint trajectories.

Figure 4 compares the proposed EAC-IK with the representative ZNN method in a synchronous bimanual trajectory-tracking task, Figure 5 illustrates the joint-trajectory profiles of both arms generated by EAC-IK, and Table 1 reports the statistical results of the evaluation metrics. As can be seen from the results, first, both methods are able to accomplish the prescribed bimanual trajectory-tracking task, with the absolute tracking errors of both arms converging rapidly. Nevertheless, EAC-IK demonstrates a better overall tracking performance and markedly improved orientation-tracking accuracy. For instance, the left-arm position tracking error of EAC-IK is $0.91\times 10^{-3}\;m$ and the left-arm orientation tracking error is $0.78\times 10^{-3}\;\mathrm{rad}$, both of which are smaller than those of ZNN ($1.90\times 10^{-3}\;m$ and $4.02\times 10^{-3}\;\mathrm{rad}$). Second, since EAC-IK explicitly incorporates the bimanual synchronization error in the modeling and automatically adjusts the convergence priority via the adaptive competition coefficient, it yields a smaller synchronization error $\varepsilon_{3}$ of $0.96\times 10^{-3}$, compared with $3.13\times 10^{-3}$ for ZNN, indicating a better synchronization performance. Finally, benefiting from the reduced-dimensional formulation, EAC-IK exhibits higher online computational efficiency. Its average runtime is 0.63ms, which is lower than the 0.82ms required by ZNN. The runtime statistics were obtained by averaging 500 repeated trials on an Intel Core i5-12500H CPU platform.

**Figure 6:**
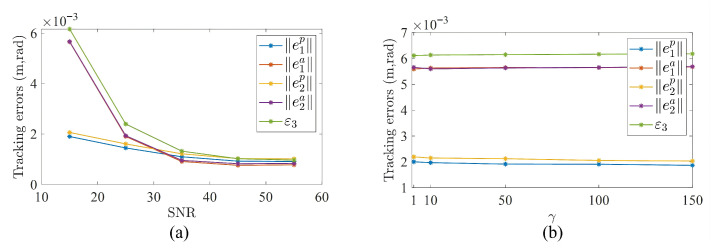
Effects on the performance of EAC-IK. (**a**) Effect of SNR. (**b**) Effect of parameter γ.

To further evaluate the performance of the proposed method, its behavior under different noise levels and parameter settings is investigated in this paper. First, tests were conducted under five different noise levels, and the corresponding results are shown in Figure 6a. The results indicate that, as the SNR decreases, both the end-effector pose tracking error and the synchronization error of EAC-IK increase. However, even under a relatively high noise level (SNR = 15), the end-effector position tracking error and orientation tracking error remain below 0.002 m and 0.006 rad, respectively, demonstrating the good noise robustness of the proposed algorithm.

Second, a parameter sensitivity study on γ was carried out. Under the condition of SNR = 15, five different values of γ were selected for testing, and the results are shown in Figure 6b. It can be observed that different values of γ have only a minor effect on the end-effector pose tracking error and synchronization error, indicating that the proposed method exhibits good robustness with respect to γ. This is because γ in the softmax competition mechanism is mainly used to adjust the sharpness of error-priority allocation, whereas the weight distribution depends more on the relative differences among the error terms. Under the current task conditions, the relative relationships among the error terms remain basically consistent, and, therefore, different values of γ do not lead to significant changes in control performance.

Overall, the proposed method establishes a reduced-dimensional inverse-kinematics solving framework based on an error-adaptive competition mechanism. Under the cost-minimization condition, the IK solver in (24) can achieve high-accuracy trajectory tracking while maintaining bimanual synchronization. Compared with existing studies, the proposed method provides higher end-effector tracking accuracy, better bimanual synchronization performance, and higher computational efficiency, making it suitable for real-time motion control of humanoid upper-limb robots.

## 5. Experimental Analysis

To verify the effectiveness and engineering practicality of the proposed EAC-IK method in practical task applications, a hyper-redundant humanoid upper-body robot was employed as the experimental platform to perform dual-arm trajectory-tracking tasks, and a comparative performance analysis with the ZNN method was conducted in realistic application scenarios. Furthermore, based on the EAC-IK method, a dual-arm cooperative transportation task was implemented on the humanoid upper-body robot, demonstrating the application potential of the proposed method in complex operational scenarios.

### 5.1. Experimental Setup

**Figure 7:**
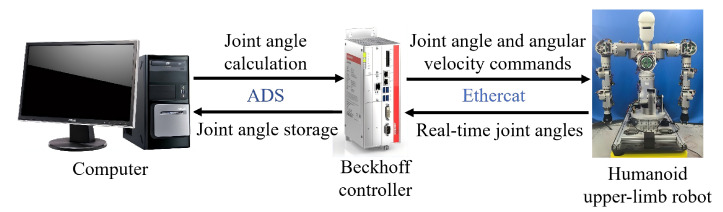
Composition of the experimental system.

Figure 7 shows the experimental system used in this study, which mainly consists of the physical humanoid upper-limb robot platform shown in Figure 3, a Beckhoff motion controller, and a host computer. The host computer is employed to run the inverse kinematics algorithm, generate joint trajectory commands, and record in real time the joint angle data fed back by the robot platform. The Beckhoff controller is responsible for sending the joint angle and joint velocity commands to the robot body. Serving as the execution platform, the humanoid upper-limb robot performs the specific experimental tasks and is used to validate the practical performance of the proposed method.

### 5.2. Experimental Results and Analysis

**Figure 8:**
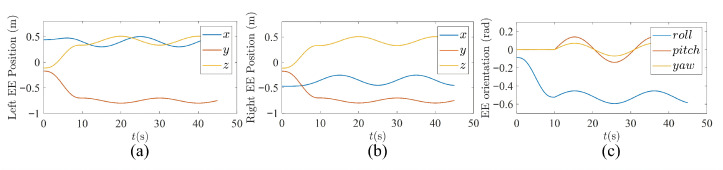
Desired end-effector pose trajectories. (**a**) Position trajectory of the left end-effector. (**b**) Position trajectory of the right end-effector. (**c**) Orientation trajectories of the left and right end-effectors.

(1) Circular trajectory-tracking task: To verify the basic performance of the proposed method in bimanual trajectory tracking, a circular trajectory-tracking experiment was conducted, since its continuous and periodic motion makes the synchronization behavior of the two arms easier to observe. The radius of the desired circular trajectory was set to 0.1m, and the normal vector of the trajectory plane was defined as ${\left[0,\;\sqrt{3}/2,\;1/2\right]}^{T}$. The trajectory centers for the left and right arms were given by ${\left[0.4,\;-0.75,\;0.42\right]}^{T}$ and ${\left[-0.3,\;-0.75,\;0.42\right]}^{T}$, respectively. Accordingly, the desired end-effector (EE) pose trajectories are shown in Figure 8. Except for the above settings, all other parameters, including the waist trajectory, were kept the same as those used in the simulations.

**Figure 9:**
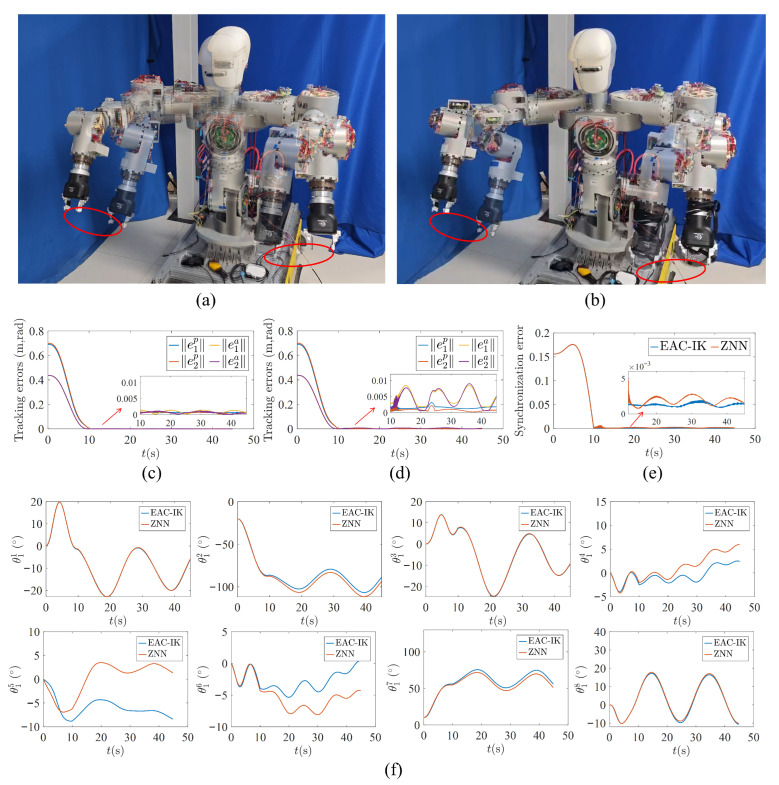
Results of the circular trajectory-tracking experiment. (**a**) Experimental snapshots based on EAC-IK, where the red circles indicate the end-effector trajectory. (**b**) Experimental snapshots based on ZNN, where the red circles indicate the end-effector trajectory. (**c**) Pose tracking errors based on EAC-IK. (**d**) Pose tracking errors based on ZNN. (**e**) Comparison of synchronization errors. (**f**) Comparison of the left-arm joint angles.

Figure 9 provides a detailed comparison between the proposed EAC-IK method and the ZNN method in the circular trajectory-tracking experiment, while Table 2 lists the corresponding statistical results of the errors. As can be seen from Figure 9a,b, both methods are capable of accomplishing the circular trajectory-tracking task effectively, with their pose tracking errors gradually converging to values close to zero, indicating that both methods possess the capability for bimanual trajectory tracking. Furthermore, as shown in Figure 9c,d, compared with ZNN, EAC-IK exhibits a superior end-effector pose-tracking performance, with smaller overall position and orientation errors, while the error magnitudes of the left and right arms remain closer to each other. For example, the position and orientation tracking errors of the left arm under EAC-IK are $0.70\times 10^{-3}\;m$ and $0.95\times 10^{-3}\;\mathrm{rad}$, respectively, both of which are smaller than those of ZNN, namely, $1.60\times 10^{-3}\;m$ and $4.72\times 10^{-3}\;\mathrm{rad}$. Meanwhile, as illustrated in Figure 9e, benefiting from the adaptive error-competition mechanism, EAC-IK achieves a smaller synchronization error with a more stable variation trend. Specifically, the synchronization error $\varepsilon_{3}$ of EAC-IK is $1.30\times 10^{-3}$, which is lower than the $1.96\times 10^{-3}$ obtained by ZNN. These results indicate that the proposed method can more effectively maintain the consistency of the error evolution of the two arms, thereby demonstrating a better bimanual synchronization performance.

(2) Bimanual object transportation task: To further verify the effectiveness of the proposed method in practical industrial scenarios, a bimanual transportation experiment involving a profile workpiece was conducted. In the experiment, the grasping points of the left and right arms were set to ${\left[0.35,\;-0.65,\;0.4\right]}^{T}$ and ${\left[-0.35,\;-0.65,\;0.4\right]}^{T}$, respectively. Under the constraint of maintaining a fixed end-effector orientation, the robot stably grasped the profile workpiece and executed a square trajectory with a side length of 0.12m.

**Figure 10:**
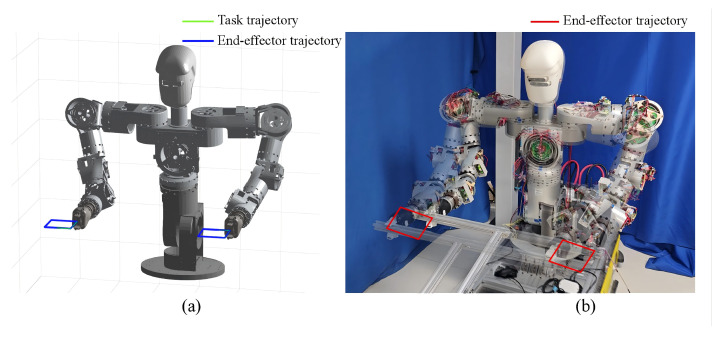
Results of the profile workpiece transportation experiment. (**a**) Three-dimensional trajectory of the robot during transportation. (**b**) Experimental snapshots of the robot.

Figure 10 shows the three-dimensional trajectory curves of this experiment, as well as the actual motion results of the robot end-effectors. It can be observed that the robot was able to stably grasp the profile workpiece throughout the entire transportation process and continuously move along the desired square trajectory, demonstrating that the proposed method has good trajectory-tracking capabilities in practical operation scenarios. This further verifies the effectiveness and practical value of EAC-IK in real-world manipulation tasks.

In summary, the circular trajectory-tracking experiment verifies that the proposed method achieves superior tracking accuracy and bimanual synchronization performance in bimanual trajectory-tracking tasks. Furthermore, the profile workpiece transportation experiment confirms its effectiveness and practical applicability in bimanual cooperative manipulation scenarios. It should be noted that the present work mainly investigates the problem of bimanual coordinated trajectory tracking from a kinematic perspective, without considering end-effector force/torque feedback. Although the proposed EAC-IK can achieve effective bimanual coordination and synchronization control, higher-precision contact tasks still require the further integration of dynamic modeling and force/torque feedback mechanisms.

## 6. Conclusions

This paper presents an EAC-IK method for dual-arm trajectory tracking of a humanoid upper-body robot. The proposed method achieves efficient reduced-dimensionality generation of dual-arm synchronous motions by constructing a unified reduced-order model of the absolute tracking errors and synchronization errors of the two arms, while incorporating an error-adaptive competition mechanism to regulate the contribution of each arm online. In addition, a virtual second-order command shaper is introduced to reconstruct and smooth the joint commands. Unlike traditional inverse kinematics methods that rely on high-dimensional Jacobian matrices, the proposed EAC-IK method simultaneously accounts for dual-arm synchronization and reduced-dimensionality solving. Simulation and experimental results on a hyper-redundant humanoid upper-body robot demonstrate that the proposed method achieves a favorable performance in terms of trajectory-tracking accuracy, dual-arm synchronization, and computational efficiency, thereby confirming its effectiveness and practicality in robotic manipulation tasks. These results suggest that the method is promising for real-time motion generation in humanoid robots performing coordinated bimanual tasks, such as object transportation and tool operation. Future work will focus on more complex task environments with obstacle avoidance and contact constraints, through the integration of end-effector force/torque feedback, the development of dynamic modeling and control for bimanual collaborative tasks, and further validation in real industrial scenarios.

## Figures and Tables

**Table 1:** Statistics of the evaluation metrics in the simulation.

Method	$∥e_{1}^{p}∥\;\left(m\right)$	$∥e_{1}^{a}∥\;\left(\mathrm{rad}\right)$	$∥e_{2}^{p}∥\;\left(m\right)$	$∥e_{2}^{a}∥\;\left(\mathrm{rad}\right)$	$\varepsilon_{3}$	Run Time (ms)
EAC-IK	$0.91\times 10^{-3}$	$0.78\times 10^{-3}$	$1.00\times 10^{-3}$	$0.83\times 10^{-3}$	$0.96\times 10^{-3}$	0.63
ZNN	$1.90\times 10^{-3}$	$4.02\times 10^{-3}$	$0.81\times 10^{-3}$	$2.58\times 10^{-3}$	$3.13\times 10^{-3}$	0.82

**Table 2:** Statistics of the evaluation metrics in the experiment.

Method	$∥e_{1}^{p}∥\;\left(m\right)$	$∥e_{1}^{a}∥\;\left(\mathrm{rad}\right)$	$∥e_{2}^{p}∥\;\left(m\right)$	$∥e_{2}^{a}∥\;\left(\mathrm{rad}\right)$	$\varepsilon_{3}$
EAC-IK	$0.70\times 10^{-3}$	$0.95\times 10^{-3}$	$0.59\times 10^{-3}$	$0.64\times 10^{-3}$	$1.30\times 10^{-3}$
ZNN	$1.60\times 10^{-3}$	$4.72\times 10^{-3}$	$0.85\times 10^{-3}$	$4.31\times 10^{-3}$	$1.96\times 10^{-3}$

## Data Availability

The original contributions presented in this study are included in the article.
